# Evaluating physicians’ knowledge, attitudes, skills and barriers regarding telemedicine in Fayoum governorate, Egypt : a cross-sectional descriptive study

**DOI:** 10.1186/s12913-025-13856-6

**Published:** 2025-12-26

**Authors:** Azza Elashiry, Wafaa Y. Abdel wahed, Huda A. EL-Kady, Shimaa Mabrouk

**Affiliations:** 1 Family Medicine Department, Faculty of Medicine, Fayoum University, Fayoum, Egypt; 2 Public Health and Community Medicine, Faculty of Medicine, Fayoum University, Fayoum, Egypt; 3 Pediatrics Department, Faculty of Medicine, Fayoum University, Fayoum, Egypt

**Keywords:** Telemedicine, Physicians, Healthcare services, Knowledge, Attitudes, Egypt

## Abstract

**Background:**

Telemedicine, characterized as the provision of healthcare services remotely, has the potential to enhance clinical management, expand service accessibility, strengthen communication among care team members, and improve the coordination of patient care.

**Objectives:**

Assess the knowledge, attitude, skills, and perceived barriers to telemedicine within medical professionals in the Fayoum Governorate.

**Methodology:**

It is a descriptive cross-sectional study performed using an online Google Form and face to face interview questionnaire. A total of 305 healthcare professionals took part in this study.

**Result:**

The mean knowledge level of our participants was 65%. The most reported barrier to telemedicine adoption was a lack of qualified personnel, cited by 91.5% of respondents. About 50% of the physicians use telemedicine daily, weekly, or monthly. Despite limited formal training (7.9%), most HCWs had basic technical skills, and comfort levels were generally neutral or low. Higher education and experience were substantial predictors of good understanding, while age and education level were key predictors of an appropriate attitude.

**Conclusions:**

The study revealed that physicians had a positive attitude towards telemedicine and showed good knowledge. Although they were highly aware of TM, the rate of TM utilization by physicians was less than satisfactory. The government must establish the necessary infrastructure and regulations to enable the implementation of telemedicine.

## Introduction

Telemedicine, a subset of telehealth, involves providing healthcare services across distances using information and communication technologies. Healthcare professionals utilize these technologies to exchange accurate information for diagnosing, treating, and preventing diseases, ultimately promoting the health of individuals and communities [1]. 

Telemedicine offers numerous advantages. It enhances patients’ access to healthcare, boosts treatment outcomes, reduces costs, and increases patient satisfaction. For healthcare providers, telemedicine saves a significant amount of time and costs and reduces travel time. Healthcare providers also show high satisfaction with telemedicine [2].

The fundamental components of telemedicine include synchronous (real-time) communication, such as video consultations; asynchronous (store and forward) communication, which allows sharing of clinical data and images for later review; remote patient monitoring through wearable devices and sensors; and mobile health (mHealth) applications that enable health promotion and self-management. Telemedicine is also seen as a solution to the shortage of healthcare professionals in developing countries [3]. 

Although telemedicine appears promising and beneficial, it still faces many challenges, particularly in developing nations. These include inadequate infrastructure in the majority of healthcare facilities, technological limitations, digital literacy gaps, financial uncertainties, data privacy concerns, and organizational resistance. Overcoming these barriers through strategic planning, investment, and strong policies is essential to fully realize telemedicine’s potential [4, 5]. 

Egypt is striving to attain Universal Health Coverage, leading to a growing demand for telehealth within standard healthcare services. However, progress in implementing telehealth has been limited, and further efforts are required to enhance the country’s telehealth preparedness. Telehealth initiatives in Egypt encounter recurring obstacles related to technology, funding, and workforce, which may impact their long-term viability. With substantial investments planned for expanding hospital capacity, Egypt has significant potential for telemedicine development [6]. 

Studies show that only about half of primary healthcare workers in Egypt are aware of telemedicine, and there are knowledge, attitude, and skill gaps among medical professionals [5, 7]. These gaps, along with infrastructural and systemic barriers, hinder the effective deployment of telemedicine services. Without an understanding of these challenges at the local level, efforts to expand telemedicine and achieve universal health coverage will fail.

This study aims to bridge this gap by assessing the knowledge, attitudes, skills, and perceived barriers to telemedicine among the physician in Fayoum Governorate.

## Methodology

### Study design and setting

This descriptive cross-sectional study assessed physicians’ knowledge, attitudes, practices, and perceived barriers toward telemedicine. The study was conducted in Fayoum Governorate, Egypt, and targeted physicians working in various healthcare facilities, including general hospitals, central hospitals, university hospitals, and primary healthcare units.

According to the **Central Agency for Public Mobilization and Statistics (CAPMAS**,** 2022)**, the population of Fayoum reached around 4.02 million people. The population is predominantly rural, with nearly 77% residing in rural areas and 23% in urban centers [8]. 

Data collection took place over eight months, from January to August 2024.

### Study population and sampling technique

The study population consisted of registered physicians in Fayoum Governorate. According to the records in Fayoum Medical Syndicate 2024, the total number of practicing physicians in the governorate is calculated at approximately 4,000.

Non-proportional Quota sampling was applied to recruit healthcare workers across the governorate, ensuring representation by healthcare facility type (primary, secondary, and tertiary care). Participants were then conveniently selected within each quota until the target numbers were achieved.

To improve participation and mitigate the limitations of online-only data collection, a hybrid data collection approach was adopted:

- Online data collection: A self-administered questionnaire was distributed via Google Forms. Access was restricted to one submission per Internet Protocol (IP) address to prevent duplicate responses.
- Face-to-face interviews: The same structured questionnaire was administered to physicians who preferred this method.

A total of 305 physicians completed the questionnaire, exceeding the minimum required sample size.

### Sample size

It was computed employing EpiCalc 2000 with the following assumptions: total physicians 4000, proportion level 50%, significance level 5%, power 80%, design effect 1, and null hypothesis value 42%. The calculated sample size was 300.

### Inclusion and exclusion criteria

All physicians actively working in Fayoum Governorate were eligible to participate. Physicians who were interns or residents in training (not yet fully licensed), or those on leave or not actively practicing during the study period, were excluded.

### Study tools

A structured questionnaire was employed to gather data. The instrument was adapted from a previously validated questionnaire developed by Jossy et al. [9] and modified to suit the local context. To ensure content validity, the questionnaire was reviewed by a panel of experts in public health, epidemiology, and telemedicine. A pilot test was conducted with 20 physicians not included in the final analysis to evaluate clarity and relevance. Based on the feedback, minor revisions were made.

The internal consistency of key sections was assessed using Cronbach’s alpha, with a reliability coefficient higher than 0.7 indicating acceptable consistency.

The final questionnaire consisted of five sections:

1. Demographic and Professional Data

- Included age, gender, place of residence, academic qualifications, years of experience, medical specialty, and type of workplace.

2. Knowledge of Telemedicine

- Comprised 11 multiple-choice items assessing physicians’ awareness of telemedicine applications. Responses were recorded as “Yes,” “No,” or “Don’t know.”
- Each correct answer was given 1 point; the maximum total score was 11.
- The knowledge score was calculated as a percentage:

Knowledge Score (%) = (Mean Score / 11) × 100.

3. Skills and Practices Related to Telemedicine

- Consisted of 7 items evaluating practical skills such as joining video consultations, setting up webcams, participating in virtual meetings, and sending documents via email.
- The total skill score was based on four items, with a maximum score of 4.

4. Attitudes Toward Telemedicine

- Included 13 items (8 related to advantages and 5 to disadvantages of telemedicine). Responses were measured on a 5-point Likert scale spanning from 1 (strongly disagree) to 5 (strongly agree).
- The total possible score ranged from 13 to 65.
- Attitude scores were converted to percentages for analysis:

Attitude Score (%) = (Mean Score / 65) × 100.

5. Perceived Barriers to Telemedicine

- Included 8 items exploring obstacles to the adoption and implementation of telemedicine services, such as lack of infrastructure and training.

### Data analysis

Data entry and statistical analyses were conducted using the Statistical Package for the Social Sciences (SPSS) software for Windows, version 27.0.1.1 (© IBM Corporation, Armonk, NY, USA). Descriptive statistics, including frequencies and percentages for categorical variables, as well as means, standard deviations, and 95% confidence intervals for continuous variables, were computed. An independent samples t-test was applied to compare knowledge and attitude scores between two groups, while one-way ANOVA was used for comparisons involving more than two groups. Pearson’s correlation coefficient was employed to analyze correlations between continuous variables. A p-value of less than 0.05 was considered statistically significant for all tests.

## Results

### Sociodemographic characteristics of healthcare workers

Table 1 demonstrates the Sociodemographic characteristics of the physicians who participated in the study. The largest proportion (40.7%) were aged 31 to 35 years. Most participants were female (73.8%), and the majority resided in urban areas (89.8%). Regarding education, 39.3% held a master’s degree, followed by those with a doctorate (28.9%). Most physicians worked in central and general hospitals (49.2%) or university hospitals (47.2%), with only 3.6% working in primary healthcare centers.

**Table 1:** Sociodemographic characteristic of physicians participating in the study

		*N*	%
**Age**	< 30	89	29.2%
	30–34	60	19.7%
	31–35	124	40.7%
	> 40	32	10.5%
Sex	Female	225	73.8%
	Male	80	26.2%
Residence	Urban	274	89.8%
	Rural	31	10.2%
Education degree	Bachelor’s	61	20.0%
	Diploma	22	7.2%
	Master’s	120	39.3%
	Doctorate	88	28.9%
	Fellowship	14	4.6%
Years of experience	Less than a year	25	8.2%
	More than ten years	56	18.4%
	Three to five years	52	17.0%
	Six to ten years	172	56.4%
Specialty	Pediatric	114	37.4%
	General medicine & specialty	97	31.8%
	General surgery& specialty	48	15.7%
	Investigational medicine	10	3.3%
	Gyn & obs	24	7.9%
	Ophthalmology	12	3.9%
Work place	University Hospitals	144	47.2%
	Central and General Hospitals	150	49.2%
	Primary Healthcare Center	11	3.6%

### Knowledge of telemedicine

Table 2 shows physicians’’ knowledge of telemedicine. Most respondents (75.7%) were aware that telemedicine allows real-time consultations, and 77.7% recognized its cost-saving benefits. However, misconceptions were also noted: 65.2% incorrectly believed that patient consent is not required if the session is recorded. The overall mean knowledge score was 7.16 ± 1.9 out of 11. The mean knowledge level of our participants was 65%, indicating a moderate level of knowledge.

**Table 2:** Assessment of physicians’ knowledge toward telemedicine

	No		Don’t know		Yes	
	*N*	%	*N*	%	*N*	%
Telemedicine can provide real-time consultations between patients and healthcare providers.	30	9.8%	44	14.4%	231	75.7%
Patient consent is not required for telemedicine consultations as long as the session is recorded.	199	65.2%	86	28.2%	20	6.6%
Telemedicine can help reduce healthcare costs by minimizing the need for in-person visits.	34	11.1%	34	11.1%	237	77.7%
A reliable internet connection is crucial for the success of telemedicine in a hospital ward.	10	3.3%	14	4.6%	281	92.1%
Telemedicine is only effective in urban areas with advanced technology infrastructure.	60	19.7%	56	18.4%	189	62.0%
Training healthcare staff in telemedicine protocols is essential for successful implementation.	10	3.3%	10	3.3%	285	93.4%
Telemedicine documentation should be comprehensive and included in the patient’s medical record.	8	2.6%	32	10.5%	265	86.9%
End-to-end encryption is essential for maintaining the security of patient data during telemedicine sessions.	8	2.6%	59	19.3%	238	78.0%
Telemedicine allows for the electronic transmission of medical records between healthcare providers.	10	3.3%	74	24.3%	221	72.5%
Patient confidentiality is less of a concern in telemedicine than in traditional in-person consultations.	203	66.6%	50	16.4%	52	17.0%
Telemedicine can only be used for remote consultations and not for patient monitoring.	105	34.4%	104	34.1%	96	31.5%
Knowledge score mean.	**7.16 ± 1.9**					

### Practices and skills in telemedicine

As shown in Table 3, only 7.9% of physicians had received formal training in telemedicine. Regarding comfort levels, 55.4% felt neutral, while 34.1% reported being uncomfortable or very uncomfortable using telemedicine.

**Table 3:** Practice and skills of physicians

		*N*	%
Have you received any training in telemedicine?	No	281	92.1%
	yes	24	7.9%
How often do you use telemedicine in your practice?	Daily	30	9.8%
	Weekly	68	22.3%
	Monthly	46	15.1%
	Rarely	138	45.2%
	Never	23	7.5%
How comfortable are you with using telemedicine technologies in your practice?	Very Uncomfortable	28	9.2%
	Uncomfortable	76	24.9%
	Neutral	169	55.4%
	Comfortable	12	3.9%
	Very Comfortable	20	6.6%
Do you know how to join a video conference?	No	82	26.9%
	Yes	223	73.1%
Do you know how to set up a webcam?	No	127	41.6%
	Yes	178	58.4%
Do you know how to participate in online discussion forums?	No	98	32.1%
	Yes	207	67.9%
Do you know how to send emails with file attachments?	No	68	22.3%
	yes	237	77.7%

### Attitudes toward telemedicine

Table 4 demonstrates physicians’ attitudes toward telemedicine. While 71.1% agreed that telemedicine reduces patient visits and 60.7% found it beneficial for provider communication, 42% strongly disagreed with its potential to reduce medical errors. Additionally, 56.4% felt that learning telemedicine was challenging, and 51.8% believed it increased workload. Out of sixty-five, the mean attitude score was 38 ± 5.7. The level of participants’ attitude was nearly 57% (moderate level of positive attitude).

**Table 4:** Attitudes of physicians toward telemedicine

	Strongly disagree		Strongly disagree		Neutral		Agree		Strongly agree	
	*N*	%	*N*	%	*N*	%	*N*	%	*N*	%
**Advantages**										
Reduce medical error.	128	42.0%	44	14.4%	97	31.8%	34	11.1%	2	0.7%
Stream lines diagnosis and treatment.	58	19.0%	6	2.0%	117	38.4%	122	40.0%	2	0.7%
Telemedicine can reduce the number of visits to healthcare centers.	2	0.7%	10	3.3%	62	20.3%	217	71.1%	14	4.6%
Boost communication among healthcare providers.	30	9.8%	6	2.0%	74	24.3%	185	60.7%	10	3.3%
Enables healthcare workers to achieve tasks more quickly.	36	11.8%	8	2.6%	124	40.7%	133	43.6%	4	1.3%
Improve clinical decisions.	86	28.2%	12	3.9%	112	36.7%	93	30.5%	2	0.7%
Deliver a more extensive healthcare service.	42	13.8%	6	2.0%	110	36.1%	141	46.2%	6	2.0%
Telemedicine is compatible with all aspects of work.	82	26.9%	16	5.2%	119	39.0%	86	28.2%	2	0.7%
**Disadvantages**										
Requires a lot of mental exertion.	38	12.5%	0	0.0%	83	27.2%	176	57.7%	8	2.6%
Learning to operate telemedicine is challenging.	36	11.8%	0	0.0%	93	30.5%	172	56.4%	4	1.3%
Increases staff workload.	26	8.5%	0	0.0%	91	29.8%	158	51.8%	30	9.8%
Increases the number of staff responsibilities.	42	13.8%	8	2.6%	54	17.7%	179	58.7%	22	7.2%
Jeopardizes information confidentiality and patient privacy.	75	24.6%	8	2.6%	90	29.5%	118	38.7%	14	4.6%

### Barriers to telemedicine implementation

Table 5 shows the barriers to telemedicine adoption. The most commonly reported challenges included a lack of qualified personnel (91.5%), time required for effective implementation (88.9%), insufficient training programs (86.2%), and infrastructure limitations (81%).

**Table 5:** Reported barriers by physicians towards implementation of telemedicine

	No		Yes	
	*N*	%	*N*	%
Lack of qualified personnel.	26	8.5%	279	91.5%
It takes time to apply the technology effectively.	34	11.1%	271	88.9%
The technology may conflict with the interests of healthcare providers.	187	61.3%	118	38.7%
Technical and operational support.	54	17.7%	251	82.3%
Financial resources.	74	24.3%	231	75.7%
Infrastructure readiness.	58	19.0%	247	81.0%
Insufficient training programs available for professionals in the field.	42	13.8%	263	86.2%
Lack of sustainable practice.	46	15.1%	259	84.9%

### Correlation between knowledge, skills, and attitudes

Table 6 shows a correlation analysis that demonstrates a strong positive relationship between knowledge and skills (*r* = 0.144, *p* = 0.012) and between knowledge and attitude (*r* = 0.243, *p* < 0.001). However, there was no association between skills and attitudes.

**Table 6:** Correlation of attitudes score with knowledge, skills, and times of practice

		Attitude score
Times of practice score	**r**	0.111
	**P value**	0.053
	**N**	305
Skills	**r**	0.144^*^
	**P value**	0.012
	**N**	305
Knowledge score	**r**	0.243^**^
	**P value**	0.000
	**N**	305

### Influence of sociodemographic factors on knowledge and attitudes

Table 7 shows the influence of sociodemographic characteristics on knowledge and attitude scores. Education level significantly affected knowledge (*p* < 0.001), with doctorate holders scoring the highest (8.05 ± 1.42). Work experience also played a role, with those having more than ten years scoring higher (7.47 ± 1.87; *p* = 0.004). Attitude scores varied by specialty, with ophthalmology specialists reporting the highest mean score (43.00 ± 8.51; *p* < 0.001).

**Table 7:** Mean knowledge and attitude scores by sociodemographic factors

		Knowledge score		P value	Attitude score		P value
		Mean	SD		Mean	SD	
Age	< 30	7.07	1.81	0.47	38.67	6.35	< 0.001 *
	30–34	7.23	1.53		35.43	3.70	
	31–35	7.27	2.03		38.10	5.75	
	> 40	6.69	2.75		40.88	5.25	
Sex	Male	7.02	2.10	0.06	37.85	5.33	0.35
	Female	7.50	1.48		38.55	6.75	
Resident	Urban	7.15	1.99	0.81	38.00	5.73	0.77
	Rural	7.06	1.79		38.32	5.87	
Education degree	Bachelor’s	6.57	1.99	< 0.001 *	38.82	4.81	0.004
	Diploma	6.00	3.09		38.27	5.40	
	Master’s	6.98	1.88		37.77	5.35	
	Doctorate	8.05	1.42		38.66	5.82	
	Fellowship	7.14	1.17		32.57	9.33	
Work place	University Hospitals	7.51	1.67	< 0.001 *	38.21	5.88	0.001
	Central and General Hospitals	6.68	2.16		37.76	5.32	
	Primary Healthcare Center	8.64	0.50		39.45	8.94	
Experience years	< 3 years	6.36	1.96	0.004	38.88	4.71	0.018
	3–5	6.57	1.61		36.57	7.02	
	6–10	7.08	2.41		36.69	5.91	
	> 10 years	7.47	1.87		38.79	5.20	
Specialty	Pediatric	6.86	2.41	0.41	38.56	3.92	< 0.001*
	General medicine & Specialty	7.33	1.72		38.29	6.84	
	General surgery& Specialty	7.38	1.84		36.33	4.57	
	Investigational medicine	6.80	1.55		32.40	7.62	
	Gyn & Obs	7.17	0.92		37.75	5.41	
	Ophthalmology	7.67	1.30		43.00	8.51	

## Discussion

This study assessed the knowledge, attitudes, skills, and perceived barriers related to telemedicine among medical professionals in Fayoum Governorate, Egypt. The findings demonstrate moderate levels of knowledge and attitude, limited hands-on experience, and persistent systemic barriers that impede effective telemedicine adoption. These results provide valuable insight into the readiness of physicians, particularly within a largely rural setting, to integrate telemedicine into routine clinical practice.

### Knowledge of telemedicine

Participants exhibited a moderate level of knowledge about telemedicine (65%), similar to findings from previous Egyptian studies by *Hasb Elnabi et al.* and *Elsaie et al.*, who documented similar awareness levels (64.4% and 69.3%, respectively) [10, 11] However, these figures remain lower than those documented in international studies from India and Australia, where knowledge levels exceeded 70% [12–14]. Differences in technological exposure, digital infrastructure, and national telemedicine integration likely account for this variation.

The positive relationship identified between knowledge and attitude in this study implies that familiarity with telemedicine enhances acceptance and willingness to adopt it. This finding aligns with previous research conducted by Hassan et al. and Naqvi et al., who reported similar associations among healthcare workers [14, 15] Participants with higher education levels and longer professional experience exhibited greater knowledge, highlighting the influence of continuous learning and exposure on digital competence. Consequently, incorporating telemedicine modules into undergraduate curricula and professional training programs could strengthen preparedness for digital healthcare delivery.

### Attitudes toward telemedicine

Overall, physicians reported moderately positive attitudes toward telemedicine (57%), recognizing its promise in improving healthcare access, reducing travel time, and enhancing cost-efficiency. These findings are consistent with previous Egyptian and regional studies that documented favorable perceptions of telemedicine’s utility and efficiency [10, 11, 15, 16]. However, nearly half of the respondents displayed neutral attitudes, suggesting limited practical experience or uncertainty about the feasibility of telemedicine in their work settings.

Attitudinal differences were noted across demographic and professional characteristics, with younger physicians and certain specialists (notably ophthalmologists) showing more favorable perceptions. This may be attributed to greater digital literacy and the adaptability of their practice to remote consultations. These variations underscore the need for tailored training approaches that account for differences in technological familiarity and clinical applicability across specialties.

Importantly, most participants emphasized the necessity of obtaining informed consent and protecting patient confidentiality during telemedicine encounters, reflecting an awareness of ethical standards. Nevertheless, some participants expressed uncertainty regarding data security and malpractice liability, concerns that have also been reported in previous Egyptian studies [11, 17, 18]. The absence of comprehensive national telemedicine regulations may contribute to these apprehensions. Thus, developing standardized legal frameworks and institutional policies that clarify responsibilities, safeguard patient data, and ensure professional protection is crucial to promote clinician confidence in telemedicine.

### Skills and practical experience

Despite broad familiarity with digital tools such as video conferencing, email, and online discussions, only 22.3% of participants reported regular telemedicine use, while nearly half used it rarely. This discrepancy between digital familiarity and actual implementation underscores a key challenge: the limited integration of telemedicine into daily clinical workflows. Similar trends have been reported in other Egyptian and international studies, indicating that infrastructural, administrative, and logistical limitations continue to restrict widespread adoption [10, 12, 16].

A noteworthy finding of this study is the strong demand for continuous training, expressed by more than 90% of respondents, which demonstrates motivation and readiness to engage in professional development related to telemedicine. However, formal training opportunities remain scarce, with the majority of participants reporting no prior structured experience. Establishing systematic, practice-oriented training programs and integrating telemedicine modules into continuing medical education could bridge this gap and facilitate competent, safe, and confident use of digital health tools.

### Perceived barriers

Participants identified several barriers to telemedicine adoption, including inadequate infrastructure (81%), financial constraints (75.7%), increased workload, and technical difficulties. These results align with earlier research highlighting similar challenges in both Egyptian and regional settings [10, 15, 19, 20]. Weak internet connectivity, lack of digital equipment, and insufficient institutional support remain persistent obstacles in resource-limited environments. The perception that telemedicine increases workload may reflect inefficiencies in workflow integration and the lack of dedicated administrative or technical assistance.

Only 28.2% of respondents believed that telemedicine was compatible with their workplace, a proportion lower than those reported in comparable studies [10, 11, 15]. This may be attributed to the predominantly rural context of Fayoum Governorate, where healthcare facilities often face infrastructural deficits and limited internet access. In rural regions, lower patient digital literacy and the absence of robust technical support may further discourage physicians from adopting telemedicine. This context should be considered when interpreting results, as it may have influenced both the practical experiences and overall attitudes of participants.

### Sociodemographic and professional influences

The study identified significant associations between higher educational attainment, longer years of experience, and greater knowledge of telemedicine. These findings are consistent with prior research demonstrating that professional maturity and continued learning enhance awareness and competence in digital healthcare [7, 10, 14, 15, 20]. Conversely, no significant associations were found between knowledge and gender or specialty, indicating that exposure and training, rather than demographic factors, primarily shape telemedicine literacy. These insights highlight the importance of continuous education programs aimed at healthcare professionals across all disciplines to ensure equitable digital readiness.

### Interpretation and clinical implications

The findings suggest that physicians in Fayoum Governorate are moderately prepared for telemedicine adoption but remain constrained by infrastructural, technical, and regulatory barriers. Positive attitudes and willingness to engage in training indicate a strong foundation for capacity-building initiatives. However, sustainable adoption will require addressing systemic limitations through investment in digital infrastructure, development of clear national policies, and incorporation of telemedicine into healthcare workflows.

Telemedicine offers an opportunity to improve healthcare accessibility, particularly in underserved and rural areas. It can reduce travel costs, mitigate workforce shortages, and enhance communication among healthcare providers. Strengthening telemedicine infrastructure and integrating it into existing healthcare systems will support progress toward universal health coverage and align with the Sustainable Development Goals.

Future research should explore telemedicine implementation in a wider range of Egyptian governorates, including urban and remote areas, to better understand geographic differences in digital readiness. Qualitative studies are also needed to explore physicians’ experiences in depth and to identify practical challenges and facilitators that cannot be captured through surveys alone. On a practical level, policymakers should prioritize expanding internet connectivity in rural healthcare facilities, establishing national telemedicine guidelines that address consent, data security, and professional liability, and integrating telemedicine into clinical workflows through targeted training programs. Evaluating the impact of structured telemedicine training and pilot implementation projects would further clarify the resources and strategies needed for sustainable digital health adoption across Egypt.

### Limitations

This study has several limitations. Because all participants were drawn from Fayoum Governorate, the findings primarily reflect the experiences of physicians working in a largely rural environment, where limited internet connectivity, lower patient digital literacy, and resource constraints may influence perceptions of telemedicine. The use of quota sampling and a combination of online and face-to-face data collection may introduce selection bias, as individuals with greater interest in or familiarity with telemedicine may have been more inclined to participate. Physicians working in primary healthcare centers were also underrepresented compared with those in secondary and tertiary facilities. Additionally, because the study employed a non-probability sampling method, the results may not be fully generalizable to all physicians in the governorate. Finally, the reliance on self-reported data raises the possibility of recall bias and social desirability bias, particularly in reporting knowledge, attitudes, and skill levels.

## Conclusion

This study assessed the knowledge, attitude, skills, and perceived barriers to telemedicine within healthcare professionals in Fayoum Governorate, Egypt. The findings revealed moderate levels of knowledge and attitude, limited hands-on experience, and several systemic and infrastructural barriers to effective implementation. Although most participants recognized the potential of telemedicine to improve healthcare accessibility, efficiency, and coordination, inadequate infrastructure, insufficient training, and the absence of standardized national regulations continue to limit its adoption.

The rural context of Fayoum Governorate likely influenced these outcomes. Limited internet connectivity, lower digital literacy among patients, and resource constraints in healthcare facilities may have shaped physicians’ experiences and perceptions of telemedicine. Nonetheless, the strong interest in acquiring further training and the overall positive orientation toward telemedicine suggest significant potential for growth if appropriate support structures are established.

To advance digital healthcare integration, it is essential to strengthen telemedicine education, improve technical infrastructure, and develop clear regulatory and ethical frameworks. Doing so will enhance healthcare professionals’ confidence and readiness to implement telemedicine safely and effectively, supporting Egypt’s progress toward equitable and sustainable health coverage.

## Data Availability

The datasets generated and/or analyzed during this study can be obtained from the corresponding author upon reasonable request.

## Abbreviations

TM: Telemedicine
HCWs: Health Care Workers
BCC: Behavior Change Communication
HCPs: Healthcare professionals
ICTs: Information computer technologies
WHO: World Health Organization
